# CpgD is a phosphoglycerate cytidylyltransferase required for ceramide diphosphoglycerate synthesis

**DOI:** 10.1016/j.jbc.2025.110386

**Published:** 2025-06-16

**Authors:** Tanisha Dhakephalkar, Ziqiang Guan, Eric A. Klein

**Affiliations:** 1 Center for Computational and Integrative Biology, Rutgers University-Camden, Camden, New Jersey, USA; 2 Department of Biochemistry, Duke University School of Medicine, Durham, North Carolina, USA; 3 Rutgers Center for Lipid Research, New Jersey Institute for Food Nutrition and Health, Rutgers University, New Brunswick, New Jersey, USA; 4 Biology Department, Rutgers University-Camden, Camden, New Jersey, USA

**Keywords:** sphingolipid, ceramide, nucleotidyltransferase, cytidylyltransferase, lipid metabolism, microbiology, enzyme kinetics

## Abstract

LPS is essential in most Gram-negative bacteria, but mutants of several species have been isolated that can survive in its absence. *Caulobacter crescentus* viability in the absence of LPS is partially dependent on the anionic sphingolipid ceramide diphosphoglycerate (CPG2). Genetic analyses showed that *ccna_01210*, which encodes a nucleotidyltransferase, is required for CPG2 production. Using purified recombinant protein, we determined that CCNA_01210 (CpgD) is a phosphoglycerate cytidylyltransferase that uses CTP and phosphoglycerate to produce CDP-glycerate, which we hypothesize is the phosphoglycerate donor for CPG2 synthesis. CpgD had optimum activity at pH 7.5 to 8 in the presence of magnesium. CpgD exhibited Michaelis-Menten kinetics concerning 3-phosphoglycerate, D-2-phosphoglycerate, and L-2-phosphoglycerate. By contrast, CTP followed Michaelis-Menten kinetics in the presence of 3-phosphoglycerate and L-2-phosphoglycerate but exhibited cooperativity with D-2-phosphoglycerate. Overall, D-2-phosphoglycerate was the preferred substrate *in vitro*. The characterization of this enzyme uncovers another step in the pathway toward CPG2 synthesis.

The outer leaflet of the outer membrane of the cell envelope of Gram-negative bacteria is primarily composed of lipopolysaccharide (LPS) (1) which provides the first line of defense against environmental stress and hydrophobic antimicrobial drugs (2). LPS is essential in most Gram-negative species, but LPS-deficient mutants have been isolated from *Acinetobacter baumanii*, *Neisseria menengitidis*, *Moraxella catarrhalis*, and *Caulobacter crescentus* (3, 4, 5, 6). While the mechanisms underlying survival in the absence of LPS appear to be species-specific, we previously showed that *C*. *crescentus* produces the anionic sphingolipid (SL) ceramide diphosphoglycerate (CPG2) which is critical for viability upon LPS depletion (6).

SLs are built upon a ceramide backbone, which consists of a sphingoid base and an N-linked fatty acid. Among the characterized bacterial SLs, these lipids can differ in acyl chain length, saturation, and branching and contain a variety of lipid headgroups. SLs play diverse roles in bacterial physiology, including host-microbe interactions (7, 8, 9), defense against bacteriophages (10), and sporulation (11). The headgroup of CPG2, which supports survival in the absence of LPS, consists of tandem phosphoglycerate moieties. Previous genetic studies have identified at least four genes that are involved in the synthesis of CPG2 (6, 12), but their specific enzymatic activities remain largely unknown. Analysis of deletion mutants shows that *ccna_01218* (*cpgB*) and *ccna_01219* (*cpgC*) are required for adding the first phosphoglycerate to form ceramide phosphoglycerate (CPG), whereas *ccna_01217* (*cpgA*) and *ccna_01210* are involved in the addition of the second phosphoglycerate to produce CPG2. In a previous report, we demonstrated that CpgB is a ceramide kinase that phosphorylates ceramide to produce ceramide 1-phosphate (C1P) (13). In the current study, we investigated the function of CCNA_01210 in producing CPG2. CCNA_01210 is annotated as a nucleotidyltransferase protein, and it shares predicted structural homology with cytidylyltransferases, which use CTP and sugar-phosphate substrates to produce CDP-sugars. The *Bacillus subtilis* cytidylyltransferase protein TagD is involved in wall teichoic acid (WTA) synthesis, where it catalyzes the transfer of glycerol-3-phosphate to CTP, forming CDP-glycerol (14). TagF then uses CDP-glycerol as a substrate to transfer phosphoglycerol to its teichoic acid membrane acceptor (15). This modification of WTA bears strong similarity to CPG2, leading to our hypothesis that CCNA_01210 uses CTP and 3-phosphoglycerate or 2-phosphoglycerate to form CDP-glycerate, which would later be used as a substrate to add a phosphoglycerate onto CPG to form CPG2. In this study, we used purified recombinant CCNA_01210 to characterize its activity and confirmed its function as a CDP-glycerate producing cytidylyltransferase.

## Results

### CCNA_01210 is required for CPG2 production

**Figure 1:**
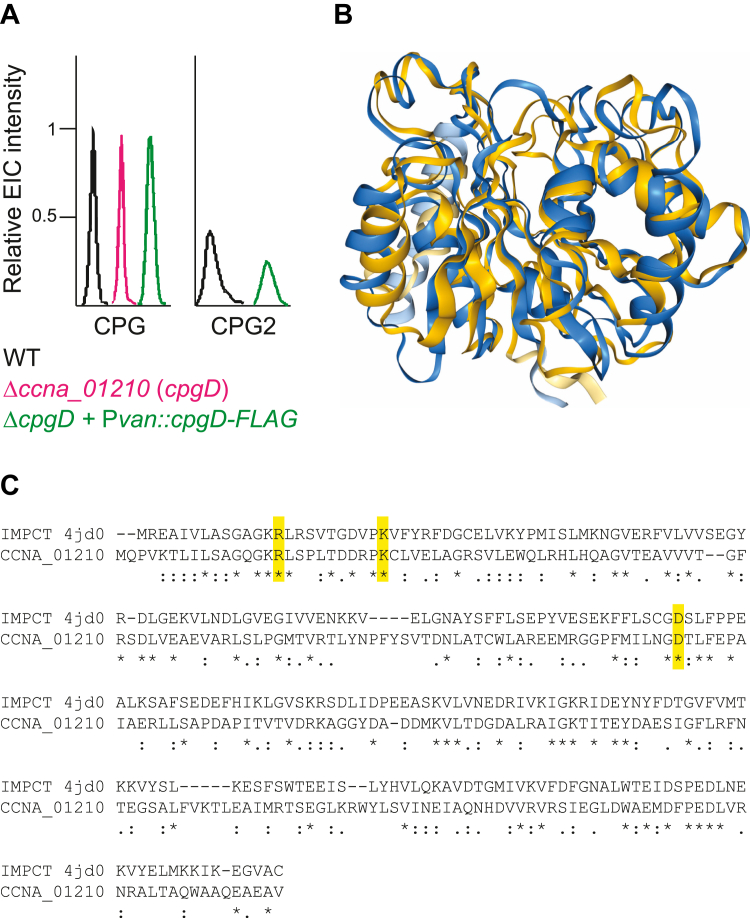
**Identification of CpgD as a putative cytidylyltransferase**. *A*, a representative Extracted-ion chromatogram (n = 2) shows the peaks corresponding to CPG and CPG2. Deletion of *ccna_01210* (*cpgD*) results in the complete loss of CPG2. CPG2 production is restored upon complementation of *cpgD*. Peak heights are normalized to CPG for each sample and offset horizontally to enhance visibility. The original EIC data is available in Figure S1. *B*, Foldseek (30) was used to generate an alignment of the Alphafold-predicted structure of CpgD (*blue*) with IMPCT (*gold*; PDB Accession 4JD0) (17). The proteins had an RMSD of 2.73 Å. *C*, the sequence alignment of CpgD and IMPCT shows conservation of three active site residues highlighted in *yellow*.

*C*. *crescentus* synthesizes two novel anionic sphingolipids, CPG and CPG2 (6). Previous genetic studies identified CpgA-C and CCNA_01210 as key enzymes for CPG2 synthesis (6, 12). Deletion of *ccna_01210* resulted in the specific loss of CPG2 while retaining CPG (Figs. 1*A* and Fig. S1) (12), suggesting that CCNA_01210 was involved in the conversion of CPG to CPG2. Complementation of the *ccna_01210* deletion restored CPG2 production (Fig. 1*A*) (12); we therefore refer to CCNA_01210 as CpgD for the remainder of this study. CpgD is annotated as a nucleotidyltransferase family protein. A structural homology search using the Alphafold-predicted structure of CpgD (16) identified *Thermotoga maritima* inositol-1-phosphate cytidylyltransferase (IMPCT; PDB 4JD0) (17) as the top hit (Fig. 1*B*). Sequence alignment showed 24% identity and 40% similarity between IMPCT and CpgD, and three critical active site residues (R16, K26, and D112) were conserved in CpgD (Fig. 1*C*) (17). IMPCT uses CTP and inositol-1-phosphate to produce CDP-inositol, an intermediate in di-myo-inositol-1,1′-phosphate (DIP) synthesis. Other members of this cytidylyltransferase family use phosphocholine (18) and phosphoglutamine (19) as substrates; we therefore hypothesized that CpgD may use phosphoglycerate to form CDP-glycerate.

### Purification and initial characterization of CpgD

**Figure 2:**
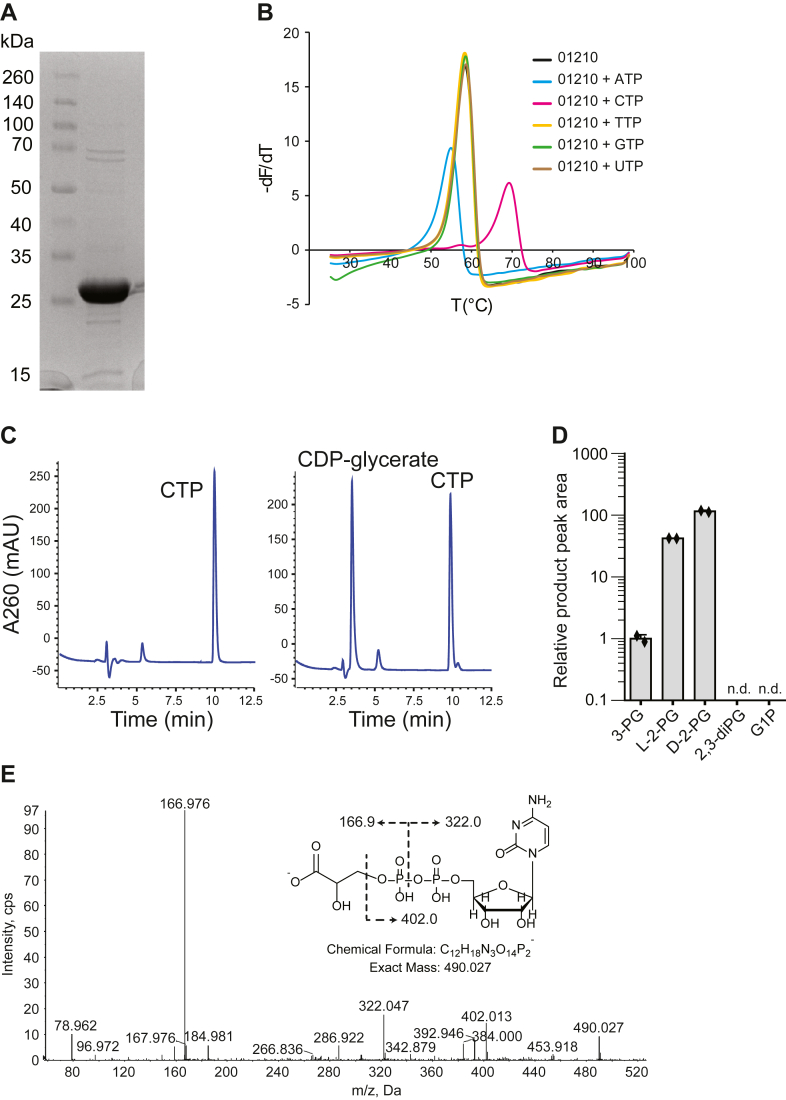
**Initial characterization of CpgD activity**. *A*, His-tagged CpgD was purified from *E*. *coli*, resolved by SDS-PAGE, and visualized by Coomassie blue staining. *B*, thermal shift assays with the indicated nucleoside triphosphates showed an increase in Tm upon the addition of CTP. (Representative graph, n = 3). *C*, HPAEC analysis of a CTP standard (*left*) or a reaction containing CpgD with CTP and 3-phosphoglycerate (*right*) shows the appearance of a peak in the reaction mixture with a retention time of 2.7 min (Representative graph, n = 2). *D*, CpgD was incubated with CTP and the indicated substrates for 3.5 h, and the products were analyzed by HPAEC. The presence of a peak at 2.7 min was indicative of a new product being formed. (2,3-diPG 2,3-diphosphoglycerate; G1P glycerol-1-phosphate; n.d. not detected; n = 2). *E*, the reaction mixture was analyzed by LC/MS in the negative ion mode to determine the identity of the reaction product. The MS/MS fragmentation products are consistent with CDP-glycerate. MS/MS analysis of the D-2-PG and L-2-PG products are available in Figure S2*B*. Reaction products were analyzed by LS/MS/MS at least twice.

We overexpressed and purified an N-terminal 6xHis-tagged CpgD from *Escherichia coli* for biochemical analysis (Fig. 2*A*). To identify potential nucleotide substrates, we performed thermal shift assays in the presence of various nucleoside triphosphates; an increase in the melting temperature of the protein was only observed upon addition of CTP, suggesting that CTP is the correct substrate (Fig. 2*B*). We note that we consistently measured a lower melting temperature in the presence of ATP, suggesting that ATP destabilizes CpgD; the significance of this observation is unclear. CpgD was incubated with CTP and either 3-phosphoglycerate (3-PG), L-2-phosphoglycerate (L-2-PG), D-2-phosphoglycerate (D-2-PG), 2,3-diphosphoglycerate, or glycerol-1-phosphate in the presence of Mg^2+,^ and the resulting nucleotide species were separated by high-performance anion-exchange chromatography (HPAEC). When comparing the reaction chromatogram to those of CTP and CDP standards, we observed a new peak with a retention time of 2.7 min in the D-2-PG, L-2-PG, and 3-PG samples; no additional peaks were observed for 2,3-diphosphoglycerate or glycerol-1-phosphate (Fig. 2, *C* and *D*). This peak was absent in a control reaction containing no enzyme, suggesting that this was the product of CpgD. Mass spectrometry analysis of this peak was consistent with CDP-glycerate (parent [M-H]^-^ ion at *m/z* 490.027), and MS/MS analysis confirmed the expected ion fragmentation (Figs. 2*E* and Fig. S2*A*).

### Effect of pH and divalent cations on CpgD activity

**Figure 3:**
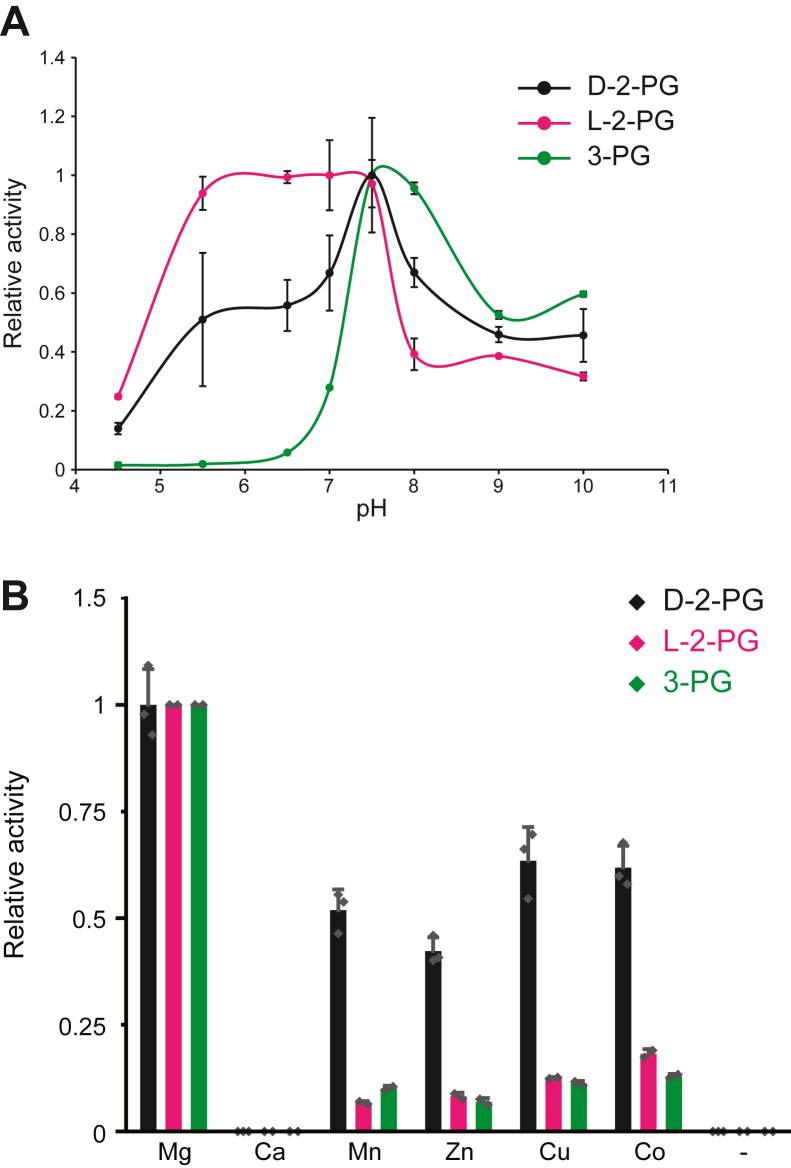
**Characterization of CpgD pH and divalent cation requirements**. *A*, CpgD activity using the indicated phosphoglycerate substrates was determined over a range of pH’s using the following buffers: acetate (pH 4.5–5.5), HEPES (pH 6.5–8.0), Tris-HCl (pH 9.0), and borate (pH 10.0). Activity was quantified by HPAEC (n = 3; *error bars* represent standard deviation). *B*, the activity of CpgD was determined in the presence of 50 mM of the indicated divalent cations. Activities were normalized to that observed with magnesium (n = 3; *error bars* represent standard deviation).

To characterize the optimal conditions for CpgD activity, we quantified CDP-glycerate production over a pH range of 4.5 to 10. While D-2-PG and 3-PG had optimal activity between pH 7.5 to 8, L-2-PG showed a broader pH range of activity (5.5–7.5) (Fig. 3*A*). We next tested CpgD activity in the presence of various divalent cations (Fig. 3*B*). The highest activity was seen in the presence of magnesium, while significant activity was also observed in the presence of copper, cobalt, zinc, or manganese. We did not observe any activity with calcium or in the absence of cations.

### Determination of CpgD kinetic parameters

**Figure 4:**
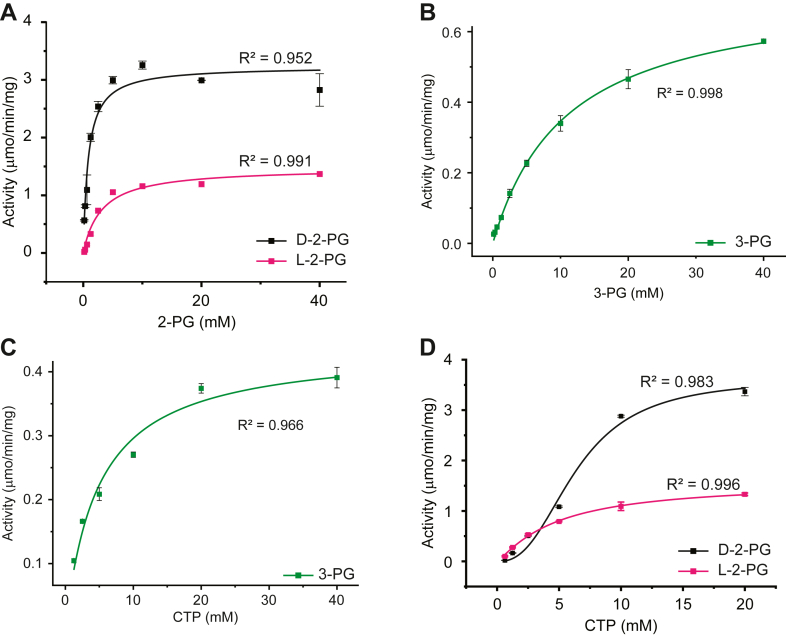
**CpgD enzyme kinetics**. The kinetic parameters of CpgD were measured by HPAEC. *A*–*D*, Michaelis-Menten kinetic parameters were determined for CpgD (n = 3, *error bars* are SD). *A*–*B*, K_m,app_ for the indicated phosphoglycerate substrate was determined by holding the CTP constant at 10 mM while varying the phosphoglycerate concentration. *C*–*D*, to determine the K_m,app_ for CTP, phosphoglycerate concentrations were held constant (10 mM) while CTP concentration varied. The curve for 3-PG was fit using the Michaelis-Menten equation, while the 2-PG curves were fit using the Hill equation. The calculated kinetic parameters are presented in Table 1.

Under the optimal pH conditions and in the presence of Mg^2+^, we measured CDP-glycerate production for 4 h to determine the linear range of activity (Fig. S2*B*). Unless otherwise specified, all kinetic studies described below were performed at 3.5 h (3-PG) or 45 min (L-2-PG and D-2-PG) in the presence of 50 mM Mg^2+^ at pH 8 (3-PG) or pH 7.5 (L-2-PG and D-2-PG). CpgD-catalyzed production of CDP-glycerate exhibited typical Michaelis–Menten kinetics with respect to all three phosphoglycerate substrates (Fig. 4, *A* and *B* and Table 1). We similarly observed Michaelis–Menten kinetics for CTP in the presence of saturating concentrations of 3-PG (Fig. 4*C*) and L-2-PG (Fig. 4*D*) but measured cooperativity with D-2-PG (Fig. 4*D*) (Table 1). The Hill coefficients for CTP were 2.5 ± 0.6 with D-2-PG and 1.2 ± 0.1 with L-2-PG.

**Table 1:** CpgD kinetic parameters.

Reaction	CTP		Phosphoglycerate	
	Vmax,app (μmol/min/mg enzyme)	Km,app (mM)	Vmax,app (μmol/min/mg enzyme)	Km,app (mM)
CTP + D-2-PG	3.6 ± 0.3	6.4 ± 0.8	3.2 ± 0.1	0.84 ± 0.16
CTP + L-2-PG	1.5 ± 0.1	6.2 ± 0.7	1.5 ± 0.1	3.1 ± 0.6
CTP + 3-PG	0.4 ± 0.03	4.8 ± 0.9	0.72 ± 0.02	10.9 ± 0.7

## Discussion

Bacteria produce sphingolipids with diverse headgroups, including sugars (10), phosphoglycerol (20), phosphoglycerate (6), and phosphoethanolamine (7). The CPG and CPG2 lipids help enable *C*. *crescentus* survival in the absence of LPS (6). Of the genes identified to play a role in CPG2 synthesis (6, 12), the only one with an ascribed function is the ceramide kinase, CpgB (13). Here, we show that CCNA_01210 (CpgD) is a cytidylyltransferase which produces CDP-glycerate, a required metabolite for CPG2 biosynthesis.

The wall teichoic acid (WTA) synthesis pathway in Gram-positive bacteria uses a similar metabolite, CDP-glycerol. These organisms use the COG0615-domain cytidylyltransferases (21) TagD or TarD to produce CDP-glycerol from CTP and phosphoglycerol (22, 23). Interestingly, despite having similar substrates and products, TagD/TarD and CpgD have no homology. Instead, CpgD exhibits both sequence and predicted structural homology to cytidylyltransferases containing the COG1213 conserved domain (21) (Fig. 1, *B* and *C*). COG1213-domain cytidylyltransferases have been reported to use phosphosugars, phosphocholine, and phosphoglutamine as substrates (16, 18, 19). Although we are not aware of any COG1213 enzymes that use phosphoglycerate as a substrate *in vivo*, the phosphoglutamine cytidylyltransferase from *Campylobacter jejuni* (Cj1416) exhibits high substrate promiscuity and can use phosphoglycerate *in vitro* when incubated with manganese rather than magnesium (19). Further structural characterization of CpgD will provide insight into the mechanism of substrate specificity among these enzymes.

Our *in vitro* assays demonstrated that the 2-PG isomers were better substrates than 3-PG, both in terms of reaction velocity and binding affinity. The Km values obtained are consistent with the measured intracellular concentrations of CTP (2.7 mM) and 3-phosphoglycerate (1.5 mM) in *E*. *coli* (24). However, data on intracellular concentrations of 2-PG in either *E*. *coli* or *C*. *crescentus* are lacking, leaving the preferred substrate *in vivo* uncertain. Since 2-PG and 3-PG are both intermediates of glycolysis, the production of CPG2 may be regulated by flux through this pathway. Alternatively, there may be a dedicated yet unidentified enzyme in *C*. *crescentus* that produces phosphoglycerate specifically for CPG2 biosynthesis.

**Figure 5:**
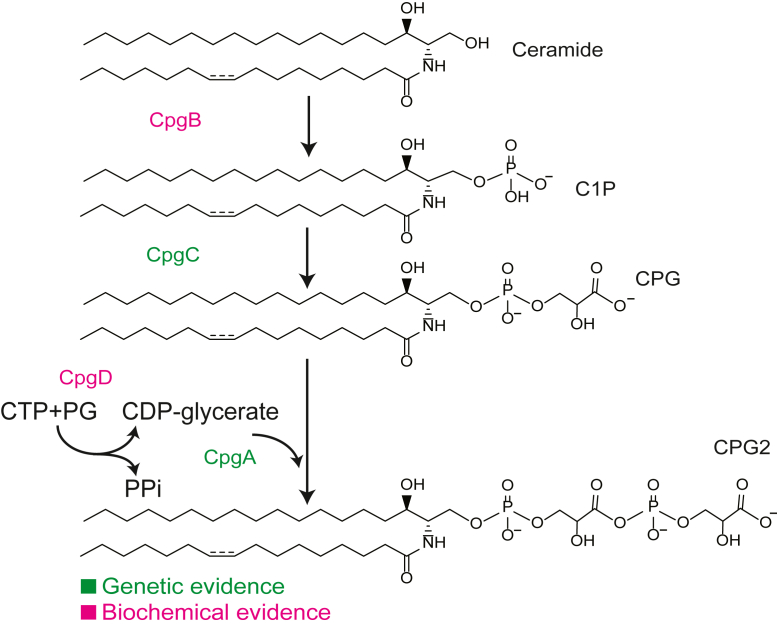
**Proposed synthetic mechanism for CPG2**. Based on previous genetic (6, 12) and biochemical (13) studies, we propose the following pathway for CPG2 synthesis. CpgB functions as a ceramide kinase that uses ATP and ceramide to produce ceramide 1-phosphate (C1P). CgpC, through a yet unidentified mechanism, converts C1P to CPG. CpgD uses CTP and phosphoglycerate to produce CDP-glycerate, which is then a substrate for CpgA to convert CPG to CPG2. Steps supported by biochemical evidence are highlighted in *magenta*, and those with genetic evidence are highlighted in *green*.

Our characterization of CpgD adds one more piece of the puzzle to the enzymatic pathway responsible for CPG2 synthesis (Fig. 5). Future work will be required to determine the activities of CpgA and CpgC, as well as to identify any other enzymes that may contribute to this biosynthetic process.

## Experimental procedures

### *C*. *crescentus* growth conditions

*C*. *crescentus* wild-type strain NA1000 and its derivatives were grown at 30 °C in peptone-yeast-extract (PYE) medium (25) for routine culturing. When necessary, antibiotics were added at the following concentrations: kanamycin 5 μg/ml in broth and 25 μg/ml in agar (abbreviated 5:25); spectinomycin 25:100. Gene expression was induced in *C*. *crescentus* with 0.25 mM vanillate.

### Deletion and complementation of ccna_01210 (cpgD)

The primers used for cloning are listed in Table 2. Δ*cpgD* was cloned by PCR amplifying the upstream (EK1698/1699) and downstream (EK1700/1701) homology fragments from NA1000 genomic DNA. The fragments were stitched together by overlapping PCR. The final purified PCR product was ligated into the EcoRI/HindIII site of pNPTS138 (MRK. Alley, unpublished). The assembled plasmid was electroporated into NA1000 followed by selection on PYE-kanamycin plates. An individual colony was grown overnight in PYE and streaked onto PYE-3% sucrose plates for *sacB* selection. Colonies were screened for the *cpgD* deletion by PCR (EK S355/S356; wild-type 1.8 kb; deletion 1.4 kb). Flag-tagged *cpgD* was amplified from NA1000 genomic DNA (EK1740/1741). The PCR product was ligated into the NdeI/NheI site of pVCFPC-1 (26). The resulting plasmid was electroporated into the Δ*cpgD* strain.

**Table 2:** Primers used in this study.

EK1698	tactgaattcGGACCTGATCGACAAGGAGA
EK1699	gatctcgttACCAGATCGCTGCGGAAG
EK1700	cgatctggtAACGAGATCGCCCAGAATC
EK1701	tactaagcttCAAGCAGCCCTGGAAGAT
EK1708	tactcatATGCAGCCGGTTAAGACCCT
EK1709	tactaagcttCTAGACCGCCTCGGCCTC
EK1740	tactcatATGCAGCCGGTTAAGACCCT
EK1741	tactgctagcTTActtgtcatcgtcatccttgtagtcGACCGCCTCGGCCTCCTG
EK S355	CGACCGATTGACCGTTCT
EK S356	GCGCACCTATCTGAAGTTCC

### Lipidomic profiling and confirmation of CPG2 production by LC/MS/MS

Lipids were extracted from *C*. *crescentus* cells using the method of Bligh and Dyer with minor modifications (27). The lipid extracts were analyzed by normal-phase LC/MS/MS in the negative ion mode as previously described (28, 29).

### Purification of His-tagged CpgD

*cpgD* was amplified (EK1708/1709) and ligated into the NdeI/HindIII site of pET-28a (EMD Millipore) to yield an N-terminal fusion protein. The resulting plasmid was transformed into *E*. *coli* strain ER2566 (New England Biolabs) for expression and purification. A 1 L culture of *E*. *coli* ER2566 cells carrying the pET28a-*cpgD* plasmid was grown in LB broth with 30 μg/ml kanamycin at 37 °C with shaking until reaching an A_600_ of 0.3. IPTG was added to a final concentration of 0.25 mM, followed by induction at 30 °C for 3 h. Cells were harvested by centrifugation at 10,000 x g. The pellet was resuspended in 25 ml of lysis buffer (50 mM NaH_2_PO_4,_ 300 mM NaCl, 10 mM imidazole) before lysing *via* two to three passages through a French press (11,000 psi). The lysate was centrifuged at 8000 x g for 10 min to remove unbroken cells, and the supernatant was passed through a 0.22 μm syringe filter. The His-tagged CpgD was purified using an ÄKTA start FPLC system and a 1 ml HisTrap HP column (Cytiva). Once the sample was loaded, it was washed with lysis buffer to remove the unbound material. Elution was carried out *via* a linear gradient of 10 to 1000 mM imidazole; 1 ml fractions were collected, and the protein elution was monitored by A_280_. Fractions that contained the purified protein were identified by SDS-PAGE and Coomassie blue staining. Fractions containing the protein were pooled and dialyzed into a buffer containing 10 mM Tris pH 8.0, 0.1 M NaCl, 2 mM EDTA, and 1 mM DTT over 36 h at 4 °C using a Slide-A-Lyzer Dialysis Cassette with a 20,000 MW cutoff (Thermo Scientific). The protein concentration was determined using the BCA Protein Assay Kit (Pierce).

### Thermal shift protein stability assay of CpgD

A 20 μl reaction was set up containing 1X GloMelt dye (Biotium), 1.2 mg/ml CpgD, 50 mM HEPES pH 7.5, 50 mM MgCl_2_, 40 nM ROX, and 10 mM nucleotide. Samples without nucleotide were used as a negative control. All reactions were carried out in triplicate. Melt curve analysis was performed using an ABI QuantStudio 6 Flex (Thermo Fisher Scientific). The protocol included an initial hold at 25 °C for 30 s, and the melt curve was measured between 25 to 99 °C with a ramp rate of 0.05 °C/s. Data were plotted using the first derivative (slope) of the fluorescence curve against temperature to calculate the Tm for each sample.

### CpgD activity assay

Enzyme activity assays for CpgD were carried out in a total volume of 20 μl containing 50 mM Tris pH 8.0 (3-phosphoglycerate) or 50 mM HEPES pH 7.5 (L-2-phosphoglycerate, and D-2-phosphoglycerate), 50 mM MgCl_2_, 10 mM CTP, and 10 mM phosphoglycerate. The reaction was initiated with the addition of 0.24 mg/ml CpgD. Reactions were stopped by heating at 90 °C for 5 min and stored at −20 °C until analysis.

### Detection of products using high-performance anion-exchange chromatography (HPAEC)

Chromatographic separation was performed using an Agilent Technologies 1200 series HPLC system equipped with a quaternary pump (G5611A), Infinity Bio-Inert HPLC Autosampler (G5667A), MWD Detector (G1365C), and OpenLAB Control Panel Software (version A.01.05). Samples were resolved on a Dionex CarboPac PA200 column (3 mm × 250 mm) with a corresponding guard column (3 mm × 50 mm) (Thermo Fisher). The column was equilibrated with 60% Buffer A (1 mM NaOH) and 40% Buffer B (1 M sodium acetate in 1 mM NaOH), and the column temperature was maintained at 30 °C. Samples were diluted 1:100 in Milli-Q water, and 25 μl was injected for HPAEC. The HPAEC run consisted of a 10 min linear gradient from 60:40 to 20:80 Buffer A: Buffer B, 2 min hold at 100% Buffer B, and 5 min of 60:40 Buffer A: Buffer B, to re-equilibrate the column. The entire run was carried out at a flow rate of 0.4 ml/min. CTP and CDP-glycerate were monitored by their absorbance at 260 nm. Product formation was measured by calculating peak areas using OpenLAB (version A.01.05). A standard curve of CTP was used to quantify product formation.

### Identification of the CpgD product

A reaction containing 50 mM Tris pH 8.0 (3-phosphoglycerate) or 50 mM HEPES pH 7.5 (L-2-phosphoglycerate, and D-2-phosphoglycerate), 50 mM MgCl_2_, 10 mM CTP, 10 mM phosphoglycerate, and 0.24 mg/ml CpgD was incubated at 35 °C for 3.5 h. For the 3-PG reaction, reverse-phase liquid chromatography-electrospray ionization/tandem mass spectrometry (LC-ESI/MS/MS) was performed using a Shimadzu LC system (comprising a solvent degasser, two LC-10A pumps, and an SCL-10A system controller) coupled to a high-resolution TripleTOF5600 mass spectrometer (AB Sciex). LC was operated at a flow rate of 200 μl/min with a linear gradient as follows: 100% of mobile phase A was held isocratically for 2 min and then linearly increased to 100% mobile phase B over 5 min and held at 100% B for 2 min. Mobile phase A was a mixture of water/acetonitrile (98/2, v/v) containing 0.1% acetic acid. Mobile phase B was a mixture of water/acetonitrile (10/90, v/v) containing 0.1% acetic acid. A Zorbax SB-C8 reversed-phase column (5 μm, 2.1 x 50 mm) was obtained from Agilent (Palo Alto, CA). The LC eluent was introduced into the ESI source of the mass spectrometer. Instrument settings for negative ion ESI/MS and MS/MS analysis of lipid species were as follows: Ion spray voltage (IS) = −4500 V; Curtain gas (CUR) = 20 psi; Ion source gas 1 (GS1) = 20 psi; De-clustering potential (DP) = −55 V; Focusing potential (FP) = −150 V. Data acquisition and analysis were performed using the Analyst TF1.5 software (AB Sciex).

For 2-PG reactions, samples were sent to the Metabolomics Core Facility of the Cancer Institute of New Jersey for analysis by liquid chromatography/mass-spectrometry (LC/MS). Briefly, reaction products were analyzed on an Orbitrap I-QX tribrid mass spectrometer (ThermoFisher Scientific) coupled to hydrophilic interaction chromatography (HILIC). Chromatography was performed on a Vanquish UHPLC system with an XBridge BEH Amide Plus column (150 mm × 2.1 mm, 2.5 μM particle size, Waters). The LC solvents were: A (95%:5% H2O: acetonitrile with 20 mM ammonium acetate, 20 mM ammonium hydroxide, pH 9.4) and B (20%:80% H2O: acetonitrile with 20 mM ammonium acetate, 20 mM ammonium hydroxide, pH 9.4). The gradient was 0 min, 100% B; 3 min, 100% B; 3.2 min, 90% B; 6.2 min, 90% B; 6.5 min, 80% B; 10.5 min, 80% B; 10.7 min, 70% B; 13.5 min, 70% B; 13.7 min, 45% B; 16 min, 45% B; 16.5 min, 100% B. The flow rate was 300 μl/min. Injection volume was 5 μl and column temperature 25 °C. The MS scans were in negative ion mode with a resolution of 70,000 at m/z 200, and the scan range was 75−1000.

### Determining the optimum pH conditions for CpgD activity

Enzyme activity assays were performed as above with the pH adjusted to 4.5, 5.5, 6.5, 7.0, 7.5, 8.0, 9.0, or 10.0. The pH was adjusted using the following buffers: acetate (pH 4.5–5.5), HEPES (pH 6.5–8.0), Tris-HCl (pH 9.0), and borate (pH 10.0). Enzyme activities were normalized to the activity at pH 8.0.

### Characterization of the divalent cation requirement for CpgD activity

Activity assays were performed as above using 50 mM of the following divalent cation salts: magnesium chloride, manganese sulfate, calcium chloride, zinc sulfate, copper sulfate, or cobalt nitrate. A control reaction was set up with no added metal ions. Enzymatic activities were normalized to those observed with MgCl_2_.

### CpgD kinetic analyses

For kinetic analyses, reactions were performed as mentioned above while varying the substrate concentrations. To determine the K_m,app_ for 3-phosphoglycerate, L-2-phosphoglycerate, and D-2-phosphoglycerate, CTP concentration was held constant (10 mM) while phosphoglycerate concentration ranged from 0.125 to 40 mM. The K_m,app_ for CTP was determined by holding the indicated phosphoglycerate concentration constant at 10 mM while varying the CTP concentration from 1.25 to 20 mM. The enzyme activity data were fit to the Michaelis–Menten or Hill equation using OriginPro (OriginLab).

## Data availability

All of the data for this work is contained within the manuscript.

## Supporting information

This article contains supporting information.

## Conflict of interest

The authors declare that they have no conflicts of interest with the contents of this article.

## References

[1] Silhavy T.J., Kahne D., Walker S. (2010). The bacterial cell envelope. Cold. Spring. Harb. Perspect. Biol..

[2] Nikaido H. (2003). Molecular basis of bacterial outer membrane permeability revisited Microbiol. Mol. Biol. Rev..

[3] Boll J.M., Crofts A.A., Peters K., Cattoir V., Vollmer W., Davies B.W. (2016). A penicillin-binding protein inhibits selection of colistin-resistant, lipooligosaccharide-deficient *Acinetobacter baumannii*. Proc. Natl. Acad. Sci. U. S. A..

[4] Peng D., Hong W., Choudhury B.P., Carlson R.W., Gu X.X. (2005). *Moraxella catarrhalis* bacterium without endotoxin, a potential vaccine candidate. Infect. Immun..

[5] Steeghs L., den Hartog R., den Boer A., Zomer B., Roholl P., van der Ley P. (1998). Meningitis bacterium is viable without endotoxin. Nature.

[6] Zik J.J., Yoon S.H., Guan Z., Stankeviciute Skidmore G., Gudoor R.R., Davies K.M. (2022). *Caulobacter* lipid A is conditionally dispensable in the absence of fur and in the presence of anionic sphingolipids. Cell. Rep..

[7] Brown E.M., Ke X., Hitchcock D., Jeanfavre S., Avila-Pacheco J., Nakata T. (2019). Bacteroides-derived sphingolipids are critical for maintaining intestinal homeostasis and symbiosis. Cell. Host. Microbe..

[8] Johnson E.L., Heaver S.L., Waters J.L., Kim B.I., Bretin A., Goodman A.L. (2020). Sphingolipids produced by gut bacteria enter host metabolic pathways impacting ceramide levels. Nat. Commun..

[9] Moye Z.D., Valiuskyte K., Dewhirst F.E., Nichols F.C., Davey M.E. (2016). Synthesis of sphingolipids impacts survival of Porphyromonas gingivalis and the presentation of surface polysaccharides. Front. Microbiol..

[10] Stankeviciute G., Guan Z., Goldfine H., Klein E.A. (2019). *Caulobacter crescentus* adapts to phosphate starvation by synthesizing anionic glycoglycerolipids and a novel glycosphingolipid. mBio.

[11] Ahrendt T., Wolff H., Bode H.B. (2015). Neutral and phospholipids of the *Myxococcus xanthus* lipodome during fruiting body formation and germination. Appl. Environ. Microbiol..

[12] Olea-Ozuna R.J., Poggio S., Bergstrom E., Osorio A., Elufisan T.O., Padilla-Gomez J. (2024). Genes required for phosphosphingolipid formation in *Caulobacter crescentus* contribute to bacterial virulence. Plos. Pathog..

[13] Dhakephalkar T., Stukey G.J., Guan Z., Carman G.M., Klein E.A. (2023). Characterization of an evolutionarily distinct bacterial ceramide kinase from *Caulobacter crescentus*. J. Biol. Chem..

[14] Fong D.H., Yim V.C., D'Elia M.A., Brown E.D., Berghuis A.M. (2006). Crystal structure of CTP:glycerol-3-phosphate cytidylyltransferase from *Staphylococcus aureus*: examination of structural basis for kinetic mechanism. Biochim. Biophys. Acta.

[15] Schertzer J.W., Brown E.D. (2003). Purified, recombinant TagF protein from *Bacillus subtilis* 168 catalyzes the polymerization of glycerol phosphate onto a membrane acceptor in vitro. J. Biol. Chem..

[16] Jumper J., Evans R., Pritzel A., Green T., Figurnov M., Ronneberger O. (2021). Highly accurate protein structure prediction with AlphaFold. Nature.

[17] Kurnasov O.V., Luk H.J., Roberts M.F., Stec B. (2013). Structure of the inositol-1-phosphate cytidylyltransferase from *Thermotoga maritima*. Acta Crystallogr. D Biol. Crystallogr..

[18] Campbell H.A., Kent C. (2001). The CTP:phosphocholine cytidylyltransferase encoded by the *licC* gene of *Streptococcus pneumoniae*: cloning, expression, purification, and characterization. Biochim. Biophys. Acta.

[19] Taylor Z.W., Raushel F.M. (2019). Manganese-induced substrate promiscuity in the reaction catalyzed by phosphoglutamine cytidylyltransferase from Campylobacter jejuni. Biochemistry.

[20] Kanzaki H., Movila A., Kayal R., Napimoga M.H., Egashira K., Dewhirst F. (2017). Phosphoglycerol dihydroceramide, a distinctive ceramide produced by *Porphyromonas gingivalis*, promotes RANKL-induced osteoclastogenesis by acting on non-muscle myosin II-A (Myh9), an osteoclast cell fusion regulatory factor. Biochim. Biophys. Acta Mol. Cell. Biol. Lipids..

[21] Wang J., Chitsaz F., Derbyshire M.K., Gonzales N.R., Gwadz M., Lu S. (2023). The conserved domain database in 2023. Nucleic. Acids. Res..

[22] D'Elia M.A., Pereira M.P., Chung Y.S., Zhao W., Chau A., Kenney T.J. (2006). Lesions in teichoic acid biosynthesis in *Staphylococcus aureus* lead to a lethal gain of function in the otherwise dispensable pathway. J. Bacteriol..

[23] Park Y.S., Sweitzer T.D., Dixon J.E., Kent C. (1993). Expression, purification, and characterization of CTP:glycerol-3-phosphate cytidylyltransferase from *Bacillus subtilis*. J. Biol. Chem..

[24] Bennett B.D., Kimball E.H., Gao M., Osterhout R., Van Dien S.J., Rabinowitz J.D. (2009). Absolute metabolite concentrations and implied enzyme active site occupancy in *Escherichia coli*. Nat. Chem. Biol..

[25] Poindexter J.S. (1964). Biological properties and classification of the *Caulobacter* group. Bacteriol. Rev..

[26] Thanbichler M., Iniesta A.A., Shapiro L. (2007). A comprehensive set of plasmids for vanillate- and xylose-inducible gene expression in *Caulobacter crescentus*. Nucleic. Acids. Res..

[27] Bligh E.G., Dyer W.J. (1959). A rapid method of total lipid extraction and purification. Can J. Biochem. Physiol..

[28] Goldfine H., Guan Z., McGenity T.J. (2015). Lipidomic Analysis of Bacteria by Thin-Layer Chromatography and Liquid Chromatography/Mass Spectrometry in Hydrocarbon and Lipid Microbiology Protocols.

[29] Guan Z., Katzianer D., Zhu J., Goldfine H. (2014). *Clostridium difficile* contains plasmalogen species of phospholipids and glycolipids. Biochim. Biophys. Acta..

[30] van Kempen M., Kim S.S., Tumescheit C., Mirdita M., Lee J., Gilchrist C.L.M. (2024). Fast and accurate protein structure search with Foldseek. Nat. Biotechnol..

