# Supplementary material for: CpgD is a phosphoglycerate cytidylyltransferase required for ceramide diphosphoglycerate synthesis
Source: J Biol Chem. 2025 Jun 16;301(7):110386. doi: 10.1016/j.jbc.2025.110386 (PMC12275187; doi:10.1016/j.jbc.2025.110386)
Supplement: Supplementary file 1 — Figure S1 [file mmc1.pdf]

Supplemental Figure 1

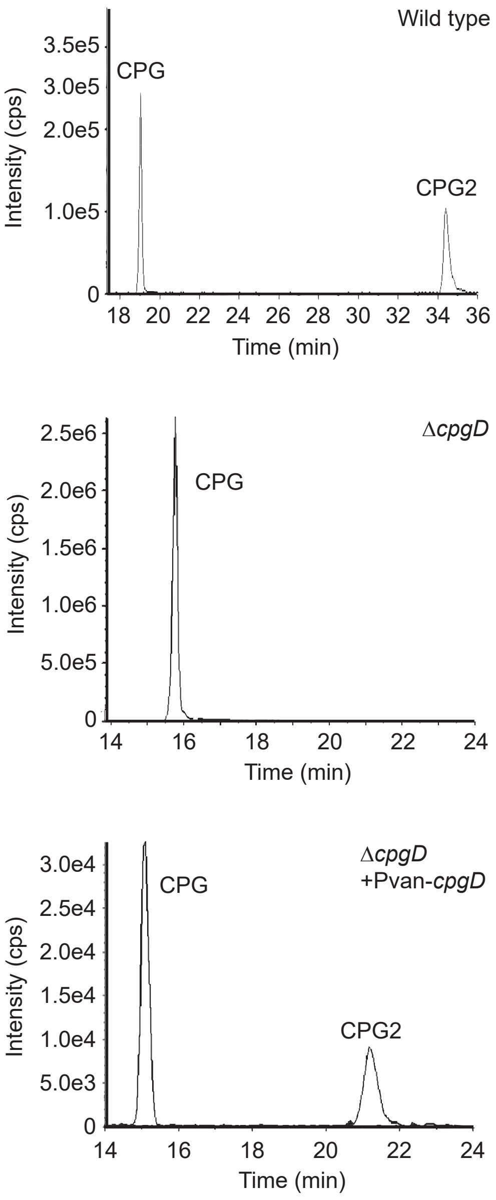

Extracted ion chromatograms. Extracted ion chromatograms (EIC) of total lipid extracts demonstrate the loss and recovery of CPG2 upon deletion and complementation of *cpgD*. These EICs correspond to the data in Figure 1A.
