# Supplementary material for: CpgD is a phosphoglycerate cytidylyltransferase required for ceramide diphosphoglycerate synthesis
Source: J Biol Chem. 2025 Jun 16;301(7):110386. doi: 10.1016/j.jbc.2025.110386 (PMC12275187; doi:10.1016/j.jbc.2025.110386)
Supplement: Figure S2 [file mmc2.pdf]

Supplemental Figure 2

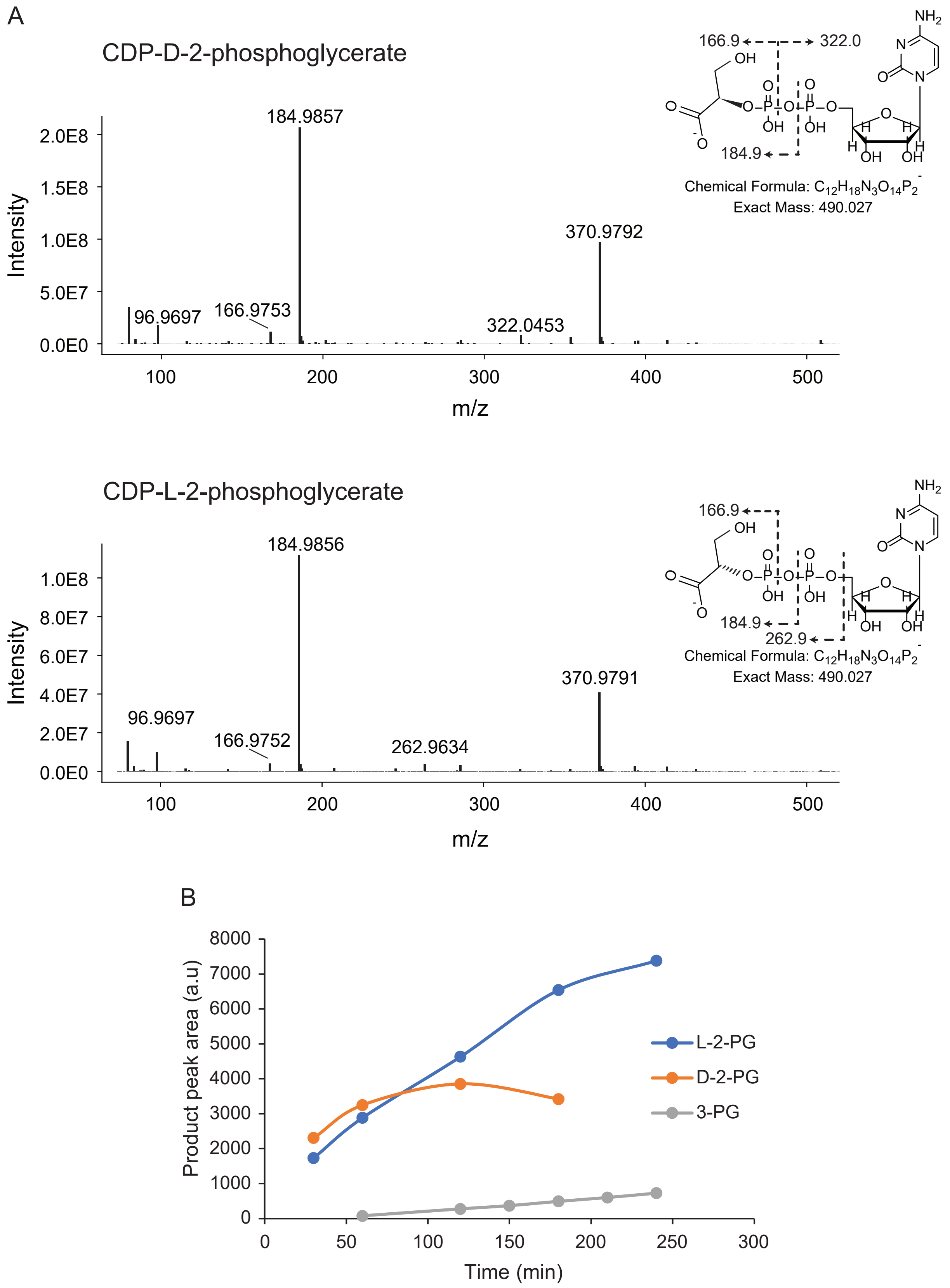

Characterization of the CpgD-mediated reaction. (A) Negative mode LC/MS/MS analysis of the D-2-PG and L-2-PG products of CpgD are consistent with the predicted fragmentation of the parent CDP-glycerate ion. (B) CpgD activity was measured as a function of time to ensure reactions were assayed within the linear range of velocity.
